# (*E*)-2-(4-*tert*-Butyl­phen­yl)-1-(4-chloro-1-ethyl-3-methyl-1*H*-pyrazol-5-yl)-2-cyano­ethenyl 2,2-dimethyl­propano­ate

**DOI:** 10.1107/S1600536810048087

**Published:** 2010-11-24

**Authors:** Man Xu, Haibo Yu, Bin Li

**Affiliations:** a Shenyang University of Chemical Technology, Shenyang 110142, People’s Republic of China; b Agrochemicals Division, Shenyang Research Institute of Chemical Industry, Shenyang 110021, People’s Republic of China

## Abstract

In the title compound, C_24_H_30_ClN_3_O_2_, the dihedral angle between the aromatic rings is 30.78 (10)°.

## Related literature

For further synthetic details, see: Kenzo *et al.* (2006 ▶); Yang *et al.* (2009 ▶). 
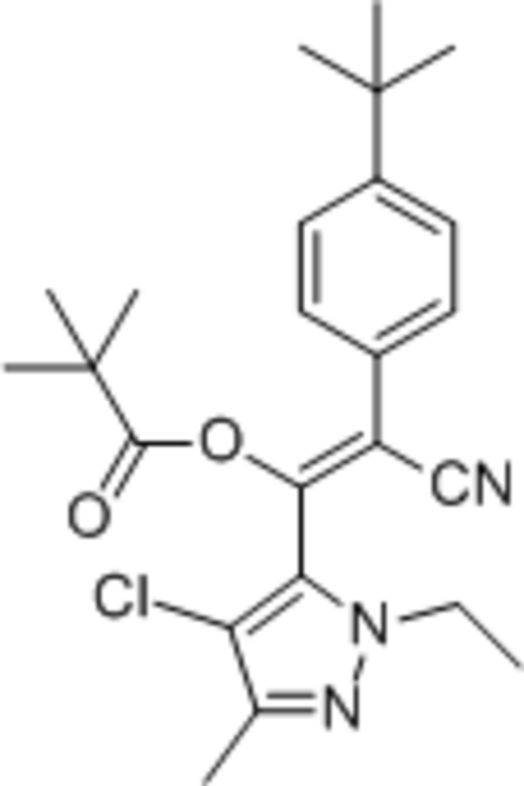


## Experimental

### 

#### Crystal data

C_24_H_30_ClN_3_O_2_
*M* *_r_* = 427.96
Monoclinic, 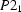

*a* = 9.997 (2) Å
*b* = 9.563 (2) Å
*c* = 12.751 (3) Å
β = 95.008 (4)°
*V* = 1214.4 (5) Å^3^
*Z* = 2
Mo *K*α radiation
μ = 0.18 mm^−1^
*T* = 296 K
0.28 × 0.22 × 0.20 mm

#### Data collection

Bruker SMART CCD diffractometer
Absorption correction: multi-scan (*SADABS*; Bruker, 2001 ▶) *T*_min_ = 0.951, *T*_max_ = 0.965
6269 measured reflections
4137 independent reflections
3802 reflections with *I* > 2σ(*I*)
*R*_int_ = 0.016

#### Refinement

*R*[*F* ^2^ > 2σ(*F* ^2^)] = 0.033
*wR*(*F* ^2^) = 0.087
*S* = 1.04
4137 reflections
279 parameters
8 restraints
H-atom parameters constrained
Δρ_max_ = 0.11 e Å^−3^
Δρ_min_ = −0.16 e Å^−3^
Absolute structure: Flack (1983 ▶), 1848 Friedel pairs
Flack parameter: 0.08 (5)

### 

Data collection: *SMART* (Bruker, 2001 ▶); cell refinement: *SAINT* (Bruker, 2001 ▶); data reduction: *SAINT*; program(s) used to solve structure: *SHELXS97* (Sheldrick, 2008 ▶); program(s) used to refine structure: *SHELXL97* (Sheldrick, 2008 ▶); molecular graphics: *SHELXTL* (Sheldrick, 2008 ▶); software used to prepare material for publication: *SHELXTL*.

## Supplementary Material

Crystal structure: contains datablocks I, global. DOI: 10.1107/S1600536810048087/hb5743sup1.cif

Structure factors: contains datablocks I. DOI: 10.1107/S1600536810048087/hb5743Isup2.hkl

Additional supplementary materials:  crystallographic information; 3D view; checkCIF report

## Figures and Tables

**Fig. 1.**
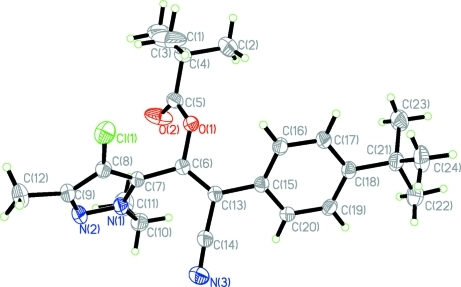
The molecular structure of (I), with 30% probability displacement ellipsoids.

## supplementary crystallographic information

### Experimental

The title compound was synthesized by 2-(4-(*tert*-butyl) phenyl)-3-(4-chloro-1-ethyl-3-methyl-1*H*-pyrazol-5-yl) -3-hydroxyacrylonitrile (Kenzo *et al.*, 2006) with pivaloyl chloride in THF. The crude products were purified by silica-gel column chromatography and then grown from heptane to afford colorless single crystals suitable for X-ray diffraction. To the mixture of 2-(4-(*tert*-butyl)phenyl)-3-(4-chloro- 1-ethyl-3-methyl-1*H*-pyrazol-5-yl)-3-hydroxyacrylonitrile (0.69 g, 2.0 mmol) and triethyl amine (0.24 g, 2.4 mmol) in THF (10 ml), pivaloyl chloride (0.29 g, 2.4 mmol) was added dropwise at roomtemperature and reacted for 1 h (Yang *et al.*, 2009). After separation through silica gel column chromatography (fluent: ethyl acetate/petroleum ether=1/20), The title product compound was gained as a white solid (0.38 g, 44%).

Anal. Calcd for C_24_H_30_Cl_1_N_3_O_2_: C, 67.35; H, 7.07; Cl, 8.28; N, 9.82; O, 7.48. Found: C, 67.33; H, 7.10; N, Cl, 8.25; N, 9.88; O, 7.44. ^1^H NMR(DMSO): 1.2 (s, 9H, CO(CH_3_)_3_), 1.34 (s, 9H, Ph-(CH_3_)_3_), 1.55 (t, 3H, CH_3_), 2.27 (s, 3H, CH_3_), 4.21 (q, 2H, N—CH_2_), 7.47 (d, 2H, Ph), 7.53 (d, 2H, Ph).

### Refinement

Although all H atoms were visible in difference maps, they werefinally placed in geometrically calculated positions, with C-Hdistances in the range 0.93–0.97 Å, andincluded in the final refinement in the riding model approximation,with *U*_iso_(H) = 1.2*U*_eq_(C) and *U*_iso_(H) = 1.5*U*_eq_(C).

### Crystal data

**Table:**

C_24_H_30_ClN_3_O_2_	*F*(000) = 456
*M**_r_* = 427.96	*D*_x_ = 1.170 Mg m^−^^3^
Monoclinic, *P*2_1_	Mo *K*α radiation, λ = 0.71073 Å
Hall symbol: P 2yb	Cell parameters from 3335 reflections
*a* = 9.997 (2) Å	θ = 2.7–25.8°
*b* = 9.563 (2) Å	µ = 0.18 mm^−^^1^
*c* = 12.751 (3) Å	*T* = 296 K
β = 95.008 (4)°	Block, colorless
*V* = 1214.4 (5) Å^3^	0.28 × 0.22 × 0.20 mm
*Z* = 2	

### Data collection

**Table:**

Bruker SMART CCD diffractometer	4137 independent reflections
Radiation source: fine-focus sealed tube	3802 reflections with *I* > 2σ(*I*)
graphite	*R*_int_ = 0.016
phi and ω scans	θ_max_ = 25.0°, θ_min_ = 1.6°
Absorption correction: multi-scan (*SADABS*; Bruker, 2001)	*h* = −11→5
*T*_min_ = 0.951, *T*_max_ = 0.965	*k* = −10→11
6269 measured reflections	*l* = −15→15

### Refinement

**Table:**

Refinement on *F*^2^	Secondary atom site location: difference Fourier map
Least-squares matrix: full	Hydrogen site location: inferred from neighbouring sites
*R*[*F*^2^ > 2σ(*F*^2^)] = 0.033	H-atom parameters constrained
*wR*(*F*^2^) = 0.087	*w* = 1/[σ^2^(*F*_o_^2^) + (0.0478*P*)^2^ + 0.1092*P*] where *P* = (*F*_o_^2^ + 2*F*_c_^2^)/3
*S* = 1.04	(Δ/σ)_max_ < 0.001
4137 reflections	Δρ_max_ = 0.11 e Å^−^^3^
279 parameters	Δρ_min_ = −0.16 e Å^−^^3^
8 restraints	Absolute structure: Flack (1983), 1848 Friedel pairs
Primary atom site location: structure-invariant direct methods	Flack parameter: 0.08 (5)

### Special details

**Table:**

Geometry. All e.s.d.'s (except the e.s.d. in the dihedral angle between two l.s. planes) are estimated using the full covariance matrix. The cell e.s.d.'s are taken into account individually in the estimation of e.s.d.'s in distances, angles and torsion angles; correlations between e.s.d.'s in cell parameters are only used when they are defined by crystal symmetry. An approximate (isotropic) treatment of cell e.s.d.'s is used for estimating e.s.d.'s involving l.s. planes.
Refinement. Refinement of *F*^2^ against ALL reflections. The weighted *R*-factor *wR* and goodness of fit *S* are based on *F*^2^, conventional *R*-factors *R* are based on *F*, with *F* set to zero for negative *F*^2^. The threshold expression of *F*^2^ > σ(*F*^2^) is used only for calculating *R*-factors(gt) *etc*. and is not relevant to the choice of reflections for refinement. *R*-factors based on *F*^2^ are statistically about twice as large as those based on *F*, and *R*- factors based on ALL data will be even larger.

### Fractional atomic coordinates and isotropic or equivalent isotropic displacement parameters (Å^2^)

**Table:**

	*x*	*y*	*z*	*U*_iso_*/*U*_eq_	
Cl1	0.89003 (6)	1.14801 (5)	0.28949 (4)	0.06542 (17)	
O1	0.69009 (13)	0.98017 (15)	0.09872 (9)	0.0502 (3)	
O2	0.59667 (17)	0.80823 (19)	0.18582 (19)	0.0901 (6)	
N1	0.92650 (17)	0.75296 (19)	0.23618 (12)	0.0542 (4)	
N2	0.99429 (19)	0.7577 (2)	0.33256 (13)	0.0651 (5)	
N3	1.1085 (2)	0.7957 (3)	0.01367 (16)	0.0828 (7)	
C1	0.5204 (3)	1.1651 (3)	0.1878 (4)	0.1185 (13)	
H1A	0.5849	1.1989	0.1423	0.178*	
H1B	0.5618	1.1578	0.2584	0.178*	
H1C	0.4461	1.2289	0.1863	0.178*	
C2	0.4021 (3)	1.0314 (4)	0.0406 (2)	0.1012 (11)	
H2A	0.4597	1.0792	−0.0040	0.152*	
H2B	0.3194	1.0821	0.0420	0.152*	
H2C	0.3838	0.9389	0.0138	0.152*	
C3	0.3704 (3)	0.9628 (4)	0.2233 (2)	0.0981 (10)	
H3A	0.2958	1.0256	0.2254	0.147*	
H3B	0.4142	0.9521	0.2929	0.147*	
H3C	0.3388	0.8734	0.1974	0.147*	
C4	0.47022 (19)	1.0221 (2)	0.15022 (16)	0.0540 (5)	
C5	0.58760 (19)	0.9229 (2)	0.15010 (15)	0.0497 (5)	
C6	0.81200 (18)	0.91176 (19)	0.10034 (14)	0.0430 (4)	
C7	0.88055 (17)	0.8811 (2)	0.20482 (13)	0.0440 (4)	
C8	0.92091 (18)	0.9714 (2)	0.28560 (14)	0.0482 (4)	
C9	0.9922 (2)	0.8913 (3)	0.36254 (15)	0.0586 (5)	
C10	0.9045 (3)	0.6173 (2)	0.18532 (18)	0.0647 (6)	
H10A	0.9902	0.5703	0.1831	0.078*	
H10B	0.8672	0.6317	0.1133	0.078*	
C11	0.8120 (3)	0.5253 (3)	0.2403 (2)	0.0935 (9)	
H11A	0.8471	0.5125	0.3122	0.140*	
H11B	0.8045	0.4362	0.2057	0.140*	
H11C	0.7250	0.5682	0.2384	0.140*	
C12	1.0620 (3)	0.9383 (4)	0.46551 (19)	0.0897 (9)	
H12A	1.1091	0.8607	0.4992	0.135*	
H12B	0.9968	0.9730	0.5101	0.135*	
H12C	1.1247	1.0112	0.4531	0.135*	
C13	0.86574 (18)	0.89023 (19)	0.00893 (13)	0.0427 (4)	
C14	1.0008 (2)	0.8375 (2)	0.01452 (15)	0.0543 (5)	
C15	0.80271 (17)	0.91367 (19)	−0.09958 (13)	0.0399 (4)	
C16	0.71135 (19)	1.0205 (2)	−0.12567 (14)	0.0479 (4)	
H16	0.6865	1.0812	−0.0738	0.058*	
C17	0.6574 (2)	1.0365 (2)	−0.22854 (14)	0.0500 (4)	
H17	0.5960	1.1081	−0.2443	0.060*	
C18	0.69157 (18)	0.9496 (2)	−0.30907 (14)	0.0447 (4)	
C19	0.78522 (18)	0.8452 (2)	−0.28222 (14)	0.0480 (4)	
H19	0.8117	0.7858	−0.3344	0.058*	
C20	0.83964 (18)	0.8279 (2)	−0.18000 (14)	0.0455 (4)	
H20	0.9023	0.7574	−0.1646	0.055*	
C21	0.6305 (2)	0.9735 (3)	−0.42226 (15)	0.0561 (5)	
C22	0.6697 (3)	0.8575 (3)	−0.49666 (18)	0.0821 (8)	
H22A	0.7654	0.8563	−0.4986	0.123*	
H22B	0.6278	0.8746	−0.5661	0.123*	
H22C	0.6402	0.7688	−0.4719	0.123*	
C23	0.4769 (2)	0.9764 (4)	−0.42351 (19)	0.0783 (7)	
H23A	0.4465	0.8918	−0.3927	0.117*	
H23B	0.4380	0.9838	−0.4948	0.117*	
H23C	0.4502	1.0553	−0.3838	0.117*	
C24	0.6812 (3)	1.1146 (3)	−0.46001 (19)	0.0822 (8)	
H24A	0.6507	1.1881	−0.4167	0.123*	
H24B	0.6471	1.1298	−0.5318	0.123*	
H24C	0.7776	1.1142	−0.4551	0.123*	

### Atomic displacement parameters (Å^2^)

**Table:**

	*U*^11^	*U*^22^	*U*^33^	*U*^12^	*U*^13^	*U*^23^
Cl1	0.0804 (4)	0.0545 (3)	0.0619 (3)	−0.0092 (3)	0.0091 (3)	−0.0059 (3)
O1	0.0546 (7)	0.0564 (8)	0.0402 (6)	0.0170 (6)	0.0082 (5)	0.0091 (6)
O2	0.0584 (9)	0.0596 (11)	0.1548 (18)	0.0126 (8)	0.0241 (10)	0.0312 (11)
N1	0.0626 (10)	0.0576 (11)	0.0410 (9)	0.0147 (8)	−0.0034 (7)	0.0008 (8)
N2	0.0675 (11)	0.0796 (14)	0.0456 (9)	0.0214 (10)	−0.0095 (8)	0.0045 (9)
N3	0.0511 (11)	0.1263 (19)	0.0710 (13)	0.0190 (12)	0.0066 (9)	0.0174 (13)
C1	0.0711 (17)	0.077 (2)	0.207 (4)	0.0204 (16)	0.009 (2)	−0.053 (2)
C2	0.0701 (16)	0.155 (3)	0.0763 (17)	0.0369 (19)	−0.0084 (13)	0.0102 (18)
C3	0.0764 (17)	0.121 (3)	0.102 (2)	0.0366 (17)	0.0362 (15)	0.0311 (19)
C4	0.0486 (10)	0.0575 (13)	0.0553 (11)	0.0138 (9)	0.0016 (8)	0.0002 (9)
C5	0.0475 (10)	0.0491 (12)	0.0516 (11)	0.0068 (8)	−0.0006 (8)	−0.0003 (9)
C6	0.0443 (9)	0.0452 (10)	0.0391 (9)	0.0031 (8)	0.0017 (7)	0.0025 (8)
C7	0.0426 (9)	0.0539 (11)	0.0355 (9)	0.0048 (8)	0.0042 (7)	0.0036 (8)
C8	0.0467 (10)	0.0565 (12)	0.0414 (10)	−0.0007 (9)	0.0042 (7)	−0.0012 (9)
C9	0.0513 (11)	0.0828 (16)	0.0402 (10)	0.0062 (11)	−0.0042 (8)	−0.0017 (10)
C10	0.0813 (15)	0.0554 (14)	0.0575 (12)	0.0162 (11)	0.0066 (10)	−0.0007 (10)
C11	0.125 (2)	0.075 (2)	0.0812 (17)	−0.0070 (17)	0.0135 (16)	0.0099 (14)
C12	0.0813 (17)	0.124 (3)	0.0584 (15)	0.0087 (16)	−0.0253 (12)	−0.0148 (15)
C13	0.0432 (8)	0.0458 (11)	0.0390 (9)	0.0012 (7)	0.0022 (7)	0.0026 (8)
C14	0.0452 (9)	0.0750 (14)	0.0428 (10)	0.0035 (9)	0.0043 (8)	0.0080 (9)
C15	0.0391 (9)	0.0426 (10)	0.0381 (9)	−0.0048 (7)	0.0047 (7)	0.0016 (8)
C16	0.0590 (11)	0.0455 (11)	0.0393 (9)	0.0045 (9)	0.0049 (8)	0.0006 (8)
C17	0.0603 (11)	0.0470 (11)	0.0426 (10)	0.0089 (9)	0.0039 (8)	0.0073 (8)
C18	0.0456 (9)	0.0498 (11)	0.0387 (9)	−0.0062 (8)	0.0035 (7)	0.0056 (7)
C19	0.0493 (10)	0.0575 (12)	0.0382 (10)	−0.0008 (9)	0.0090 (8)	−0.0047 (8)
C20	0.0416 (9)	0.0523 (11)	0.0431 (10)	0.0067 (8)	0.0061 (7)	0.0038 (8)
C21	0.0568 (11)	0.0720 (14)	0.0393 (10)	−0.0041 (10)	0.0024 (8)	0.0033 (10)
C22	0.0937 (18)	0.106 (2)	0.0452 (13)	0.0079 (16)	−0.0039 (12)	−0.0117 (13)
C23	0.0596 (13)	0.112 (2)	0.0608 (13)	−0.0039 (14)	−0.0105 (10)	0.0039 (14)
C24	0.0965 (18)	0.096 (2)	0.0527 (13)	−0.0131 (15)	−0.0027 (12)	0.0278 (13)

### Geometric parameters (Å, °)

**Table:**

Cl1—C8	1.718 (2)	C11—H11B	0.9600
O1—C5	1.377 (2)	C11—H11C	0.9600
O1—C6	1.382 (2)	C12—H12A	0.9600
O2—C5	1.187 (3)	C12—H12B	0.9600
N1—N2	1.351 (2)	C12—H12C	0.9600
N1—C7	1.356 (3)	C13—C14	1.437 (3)
N1—C10	1.459 (3)	C13—C15	1.486 (2)
N2—C9	1.334 (3)	C15—C20	1.388 (3)
N3—C14	1.149 (3)	C15—C16	1.391 (3)
C1—C4	1.519 (4)	C16—C17	1.382 (2)
C1—H1A	0.9600	C16—H16	0.9300
C1—H1B	0.9600	C17—C18	1.387 (3)
C1—H1C	0.9600	C17—H17	0.9300
C2—C4	1.503 (3)	C18—C19	1.391 (3)
C2—H2A	0.9600	C18—C21	1.534 (3)
C2—H2B	0.9600	C19—C20	1.378 (3)
C2—H2C	0.9600	C19—H19	0.9300
C3—C4	1.532 (3)	C20—H20	0.9300
C3—H3A	0.9600	C21—C22	1.533 (4)
C3—H3B	0.9600	C21—C24	1.534 (3)
C3—H3C	0.9600	C21—C23	1.534 (3)
C4—C5	1.509 (3)	C22—H22A	0.9600
C6—C13	1.341 (3)	C22—H22B	0.9600
C6—C7	1.473 (2)	C22—H22C	0.9600
C7—C8	1.378 (3)	C23—H23A	0.9600
C8—C9	1.391 (3)	C23—H23B	0.9600
C9—C12	1.501 (3)	C23—H23C	0.9600
C10—C11	1.495 (4)	C24—H24A	0.9600
C10—H10A	0.9700	C24—H24B	0.9600
C10—H10B	0.9700	C24—H24C	0.9600
C11—H11A	0.9600		
			
C5—O1—C6	119.76 (15)	H11A—C11—H11C	109.5
N2—N1—C7	111.58 (17)	H11B—C11—H11C	109.5
N2—N1—C10	118.59 (18)	C9—C12—H12A	109.5
C7—N1—C10	129.66 (16)	C9—C12—H12B	109.5
C9—N2—N1	105.88 (17)	H12A—C12—H12B	109.5
C4—C1—H1A	109.5	C9—C12—H12C	109.5
C4—C1—H1B	109.5	H12A—C12—H12C	109.5
H1A—C1—H1B	109.5	H12B—C12—H12C	109.5
C4—C1—H1C	109.5	C6—C13—C14	117.17 (16)
H1A—C1—H1C	109.5	C6—C13—C15	128.05 (16)
H1B—C1—H1C	109.5	C14—C13—C15	114.78 (15)
C4—C2—H2A	109.5	N3—C14—C13	176.6 (2)
C4—C2—H2B	109.5	C20—C15—C16	117.94 (16)
H2A—C2—H2B	109.5	C20—C15—C13	118.69 (16)
C4—C2—H2C	109.5	C16—C15—C13	123.34 (16)
H2A—C2—H2C	109.5	C17—C16—C15	120.12 (17)
H2B—C2—H2C	109.5	C17—C16—H16	119.9
C4—C3—H3A	109.5	C15—C16—H16	119.9
C4—C3—H3B	109.5	C16—C17—C18	122.38 (17)
H3A—C3—H3B	109.5	C16—C17—H17	118.8
C4—C3—H3C	109.5	C18—C17—H17	118.8
H3A—C3—H3C	109.5	C17—C18—C19	116.85 (16)
H3B—C3—H3C	109.5	C17—C18—C21	120.26 (18)
C2—C4—C5	108.99 (18)	C19—C18—C21	122.86 (17)
C2—C4—C1	110.5 (3)	C20—C19—C18	121.39 (17)
C5—C4—C1	109.33 (18)	C20—C19—H19	119.3
C2—C4—C3	108.8 (2)	C18—C19—H19	119.3
C5—C4—C3	108.39 (19)	C19—C20—C15	121.29 (17)
C1—C4—C3	110.9 (2)	C19—C20—H20	119.4
O2—C5—O1	120.98 (18)	C15—C20—H20	119.4
O2—C5—C4	127.8 (2)	C22—C21—C24	109.5 (2)
O1—C5—C4	111.17 (17)	C22—C21—C18	111.85 (19)
C13—C6—O1	118.64 (15)	C24—C21—C18	108.12 (17)
C13—C6—C7	124.41 (16)	C22—C21—C23	108.4 (2)
O1—C6—C7	116.61 (15)	C24—C21—C23	109.8 (2)
N1—C7—C8	106.06 (16)	C18—C21—C23	109.15 (16)
N1—C7—C6	124.43 (17)	C21—C22—H22A	109.5
C8—C7—C6	129.32 (18)	C21—C22—H22B	109.5
C7—C8—C9	106.28 (19)	H22A—C22—H22B	109.5
C7—C8—Cl1	126.62 (15)	C21—C22—H22C	109.5
C9—C8—Cl1	127.10 (16)	H22A—C22—H22C	109.5
N2—C9—C8	110.19 (17)	H22B—C22—H22C	109.5
N2—C9—C12	121.3 (2)	C21—C23—H23A	109.5
C8—C9—C12	128.5 (2)	C21—C23—H23B	109.5
N1—C10—C11	113.0 (2)	H23A—C23—H23B	109.5
N1—C10—H10A	109.0	C21—C23—H23C	109.5
C11—C10—H10A	109.0	H23A—C23—H23C	109.5
N1—C10—H10B	109.0	H23B—C23—H23C	109.5
C11—C10—H10B	109.0	C21—C24—H24A	109.5
H10A—C10—H10B	107.8	C21—C24—H24B	109.5
C10—C11—H11A	109.5	H24A—C24—H24B	109.5
C10—C11—H11B	109.5	C21—C24—H24C	109.5
H11A—C11—H11B	109.5	H24A—C24—H24C	109.5
C10—C11—H11C	109.5	H24B—C24—H24C	109.5
			
C7—N1—N2—C9	−0.5 (2)	Cl1—C8—C9—C12	−1.7 (3)
C10—N1—N2—C9	−176.2 (2)	N2—N1—C10—C11	67.3 (3)
C6—O1—C5—O2	−8.6 (3)	C7—N1—C10—C11	−107.5 (3)
C6—O1—C5—C4	172.86 (15)	O1—C6—C13—C14	171.17 (17)
C2—C4—C5—O2	−107.5 (3)	C7—C6—C13—C14	−1.9 (3)
C1—C4—C5—O2	131.6 (3)	O1—C6—C13—C15	−9.1 (3)
C3—C4—C5—O2	10.7 (3)	C7—C6—C13—C15	177.85 (18)
C2—C4—C5—O1	70.9 (2)	C6—C13—C14—N3	−178 (100)
C1—C4—C5—O1	−49.9 (3)	C15—C13—C14—N3	2(4)
C3—C4—C5—O1	−170.9 (2)	C6—C13—C15—C20	−148.6 (2)
C5—O1—C6—C13	129.59 (19)	C14—C13—C15—C20	31.2 (2)
C5—O1—C6—C7	−56.8 (2)	C6—C13—C15—C16	33.6 (3)
N2—N1—C7—C8	−0.2 (2)	C14—C13—C15—C16	−146.64 (19)
C10—N1—C7—C8	174.9 (2)	C20—C15—C16—C17	1.7 (3)
N2—N1—C7—C6	175.15 (17)	C13—C15—C16—C17	179.58 (17)
C10—N1—C7—C6	−9.7 (3)	C15—C16—C17—C18	−0.5 (3)
C13—C6—C7—N1	−58.8 (3)	C16—C17—C18—C19	−0.9 (3)
O1—C6—C7—N1	128.03 (19)	C16—C17—C18—C21	−178.96 (18)
C13—C6—C7—C8	115.5 (2)	C17—C18—C19—C20	1.0 (3)
O1—C6—C7—C8	−57.7 (3)	C21—C18—C19—C20	178.99 (17)
N1—C7—C8—C9	0.8 (2)	C18—C19—C20—C15	0.3 (3)
C6—C7—C8—C9	−174.29 (17)	C16—C15—C20—C19	−1.7 (3)
N1—C7—C8—Cl1	−179.68 (14)	C13—C15—C20—C19	−179.61 (17)
C6—C7—C8—Cl1	5.3 (3)	C17—C18—C21—C22	−174.0 (2)
N1—N2—C9—C8	1.0 (2)	C19—C18—C21—C22	8.1 (3)
N1—N2—C9—C12	−178.1 (2)	C17—C18—C21—C24	65.4 (2)
C7—C8—C9—N2	−1.1 (2)	C19—C18—C21—C24	−112.5 (2)
Cl1—C8—C9—N2	179.36 (16)	C17—C18—C21—C23	−54.0 (3)
C7—C8—C9—C12	177.9 (2)	C19—C18—C21—C23	128.0 (2)
